# Change in multimodal MRI markers predicts dementia risk in cerebral small vessel disease

**DOI:** 10.1212/WNL.0000000000004594

**Published:** 2017-10-31

**Authors:** Eva A. Zeestraten, Andrew J. Lawrence, Christian Lambert, Philip Benjamin, Rebecca L. Brookes, Andrew D. Mackinnon, Robin G. Morris, Thomas R. Barrick, Hugh S. Markus

**Affiliations:** From the Neuroscience Research Centre (E.A.Z., C.L., P.B., T.R.B.), Cardiovascular and Cell Sciences Research Institute, St George's University of London; Stroke Research Group (A.J.L., R.L.B., H.S.M.), Clinical Neurosciences, University of Cambridge; Atkinson Morley Regional Neuroscience Centre (A.D.M.), St George's NHS Healthcare Trust; and Department of Psychology (R.G.M.), King's College Institute of Psychiatry, Psychology, and Neuroscience, London, UK.

## Abstract

**Objective:**

To determine whether MRI markers, including diffusion tensor imaging (DTI), can predict cognitive decline and dementia in patients with cerebral small vessel disease (SVD).

**Methods:**

In the prospective St George's Cognition and Neuroimaging in Stroke study, multimodal MRI was performed annually for 3 years and cognitive assessments annually for 5 years in a cohort of 99 patients with SVD, defined as symptomatic lacunar stroke and confluent white matter hyperintensities (WMH). Progression to dementia was determined in all patients. Progression of WMH, brain volume, lacunes, cerebral microbleeds, and a DTI measure (the normalized peak height of the mean diffusivity histogram distribution) as a marker of white matter microstructural damage were determined.

**Results:**

Over 5 years of follow-up, 18 patients (18.2%) progressed to dementia. A significant change in all MRI markers, representing deterioration, was observed. The presence of new lacunes, and rate of increase in white matter microstructural damage on DTI, correlated with both decline in executive function and global functioning. Growth of WMH and deterioration of white matter microstructure on DTI predicted progression to dementia. A model including change in MRI variables together with their baseline values correctly classified progression to dementia with a C statistic of 0.85.

**Conclusions:**

This longitudinal prospective study provides evidence that change in MRI measures including DTI, over time durations during which cognitive change is not detectable, predicts cognitive decline and progression to dementia. It supports the use of MRI measures, including DTI, as useful surrogate biomarkers to monitor disease and assess therapeutic interventions.

Cerebral small vessel disease (SVD) is the major pathology underlying vascular dementia and an important cause of age-related cognitive decline.^1^ While many elderly patients develop radiologic signs of SVD,^2^ only a minority progress to dementia. Better methods are required to identify the subgroup who rapidly decline.

Using rate of cognitive decline as a predictive tool in SVD has been shown to be limited^3^ due to the slow rate of decline^4^ and insensitivity of cognitive tests to change. Research has therefore focused on using MRI features of SVD, such as lacunes, white matter hyperintensities (WMH), and brain atrophy,^5,6^ as surrogate markers.^7^ Diffusion tensor imaging (DTI) is of particular interest; it is highly sensitive to white matter (WM) microstructural damage in SVD and demonstrates widespread abnormalities in the apparently normal-appearing WM (NAWM).^8^

Cross-sectional studies have shown that both lacunes and diffuse WM damage detected on DTI are associated with cognitive impairment.^9–12^ It has been hypothesized that both result in white matter track disruption, and disconnection of distributed networks underlying executive function (EF) and processing speed (PS), the 2 domains most affected in SVD.^13,14^ However, almost all previous data are cross-sectional and therefore give information only on association, not prediction. The longitudinal St George's Cognition and Neuroimaging in Stroke (SCANS) study was established to determine whether change in multimodal MRI, including DTI, predicts cognitive decline and dementia in SVD.

## METHODS

### Patients.

Details of the SCANS study have been published previously.^7,10^ In brief, patients presenting with symptomatic SVD, defined as a clinical lacunar stroke syndrome^15^ with MRI evidence of an anatomically corresponding lacunar infarct, and with confluent regions of WMH graded ≥2 on the modified Fazekas scale,^16^ were enrolled from 3 stroke services covering a geographically contiguous region in South London. Patients underwent MRI annually for 3 years and cognitive testing annually for 5 years. At each visit, repeat recordings of cardiovascular risk factors and blood pressure were performed. Data on progression to dementia were collected during follow-up and from hospital and family doctor records.

#### Standard protocol approvals, registrations, and patient consents.

The study was approved by the Wandsworth (London) research ethics committee and all patients provided written informed consent. The study is registered with the UK Clinical Research Network (public.ukcrn.org.uk/; study ID 4577).

#### Available data.

A total of 121 patients were recruited. Of these, 103 attended more than one cognitive assessment. Eighteen patients only attended one assessment due to death (n = 7), study withdrawal (n = 6), house move (n = 1), lost to follow-up (n = 2), or withdrawal from full neuropsychological testing (n = 2). Of the 103 who attended cognitive assessments more than once, MRI data at multiple time points were available for 99; 4 withdrew from imaging but remained in the study for neuropsychological testing. In this analysis, we describe the relationship between change of MRI measures and both cognitive change and progression to dementia in all 99 who had at least one follow-up MRI. The number of complete MRI and cognitive assessments at each time point are shown in table e-1 at Neurology.org.

Demographic characteristics of the 99 patients (table 1) who attended one or more follow-up MRI and cognitive sessions compared to the 22 who did not have been described previously.^4^ There were no significant differences in any imaging measures between the groups, but patients who remained in the study were younger and had higher baseline Mini-Mental State Examination (MMSE) scores. At study entry, no patients had had intracerebral hemorrhage, had superficial siderosis, or met the modified Boston criteria for definite, probable, or possible cerebral amyloid angiopathy.^17^

**Table 1:**
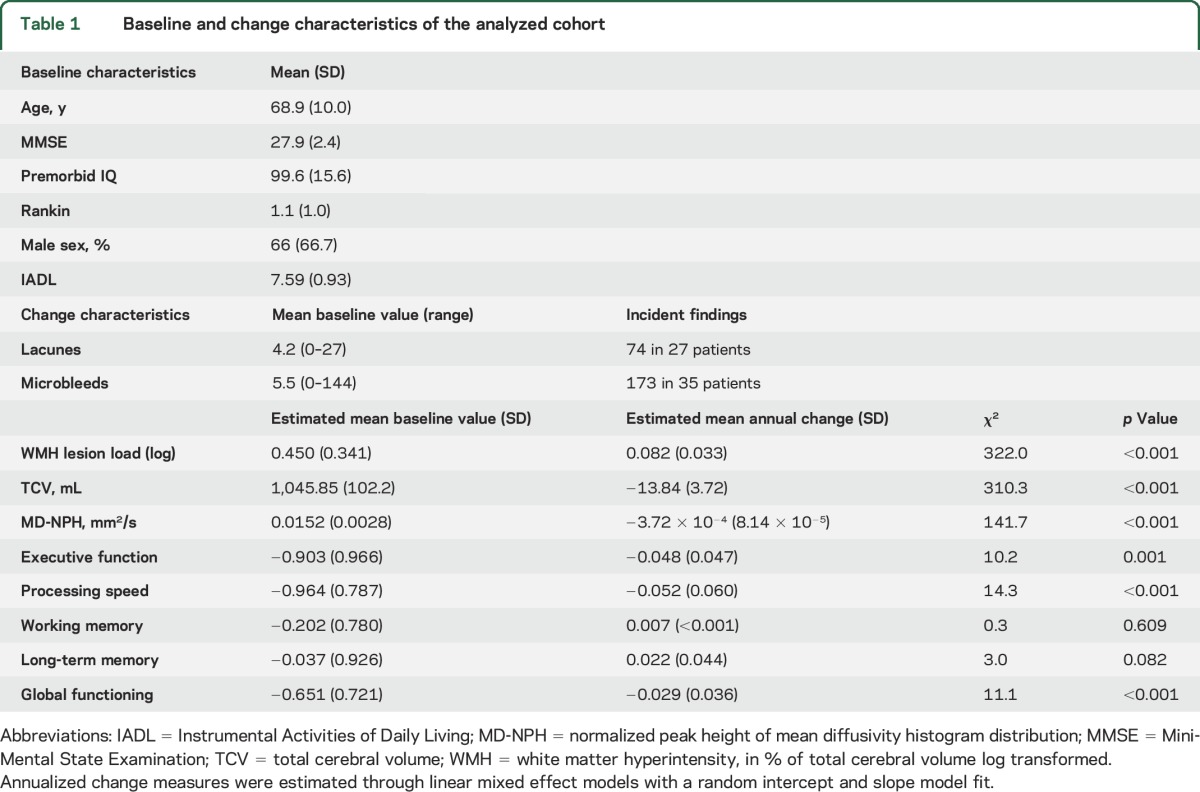
Baseline and change characteristics of the analyzed cohort

### MRI acquisition.

MRI at baseline and each follow-up visit were acquired using the same 1.5T GE Signa HDxt system (General Electric, Milwaukee, WI) using identical image acquisition protocols as previously published^10^ and described in appendix e-1.

### MRI analysis.

#### Structural preprocessing.

A longitudinal tissue segmentation pipeline optimized to our SVD cohort, described in detail in a previous publication,^18^ was performed to obtain segmentations for gray matter, NAWM, WMH, and CSF based on coregistered fluid-attenuated inversion recovery and T1-weighted images.

#### Conventional MRI markers of SVD.

The tissue segmentation maps were used to calculate total cerebral volume and WMH lesion load (log-transformed to adjust for its skewed distribution). Lacunes and cerebral microbleeds (CMB) were identified by a trained independent rater according to agreed neuroimaging standards.^6,19^ Detailed calculations and descriptions for these MRI markers are shown in appendix e-2.

#### Diffusion preprocessing.

Detailed preprocessing steps of diffusion-weighted images have been described elsewhere^20^ and in appendix e-3. Preprocessing was applied to evaluate the normalized mean diffusivity (MD) histogram distributions of all WM tissue (i.e., NAWM plus WMH) (range 0–0.004 mm^2^/s, bin width 0.000004 mm^2^/s). The normalized frequency of voxels with the histogram peak value in all WM tissue, the MD normalized peak height (MD-NPH) (figure e-1), was used as a measure of tissue microstructure over time as we have previously shown it is the most stable and sensitive DTI measure of change.^20^

### Cognitive assessment.

#### Cognitive index scores.

A battery of well-established, standardized tasks sensitive to the cognitive impairments seen in SVD was carried out annually. Full details have been published previously.^10^ In brief, cognitive tasks (table e-2) were age-scaled using published normative data, converted to *z* scores, and grouped into broad cognitive domains. Averaging across component scores within each cognitive domain created cognitive index scores for EF, PS, working memory, and long-term memory. An overall global functioning score based on all administered tests was produced. In addition, premorbid intelligence was assessed.

#### Dementia.

Information on conversion to dementia was available for all 99 patients. Dementia was diagnosed using the DSM-5^21^ definition of major neurocognitive disorder, and was present if individuals met one of the following criteria:

1. A diagnosis of dementia made in a memory clinic or equivalent clinical service
2. After review of medical records and cognitive assessments by a neurologist and clinical neuropsychologist, both blinded to MRI and risk factor information, who agreed that the clinical picture met DSM-5 criteria
3. An MMSE score consistently <24, indicative of cognitive impairment,^22^ and reduced capabilities in daily living as measured by a score ≤7 on the Instrumental Activities of Daily Living (IADL)^23^

The presence of dementia was determined before comparison of cognitive and MRI data. Date of dementia onset was defined as the date of diagnosis. If no exact date was known and dementia conversion was based on review of patient data or cognitive performance, the midpoint date between the visit at which the diagnosis was established and the previous visit was used.

### Statistical analyses.

We employed linear mixed effect (LME) models to estimate annualized change rates in MRI and cognitive markers based on all available time points. MRI and cognitive indices data were modeled separately in MLwiN 2.1 (Centre for Multilevel Modelling, University of Bristol).^24^ Intercepts and linear trajectories (i.e., annual change rate) across the follow-up period as a function of time were allowed to vary with fixed and random effects. The presence of detectable change was assessed on the basis of significance of the average fixed effect slope of time evaluated using a Wald test. Slopes for each patient as estimated by the LME models were extracted and used for further analyses.

First, univariate linear regression analyses were performed between annualized cognitive change rates and MRI markers using SPSS 22.0 (IBM Corp., Armonk, NY). Lacunes and CMB were treated as binary variables (i.e., no change or any new lesions), due to the low frequency of new lesions. Multivariate stepwise linear regression analysis was used to investigate the relationship between annualized change rates of different MRI markers with cognitive decline. Baseline age, premorbid IQ, and sex were added as covariates.

Predictive abilities of MRI change rates and vascular risk factors for dementia conversion were assessed by univariate Cox regression. LME estimates of change in MRI variables for these dementia models were recalculated to exclude MRI data acquired after conversion to dementia (n = 3 participants, n = 3 observations). This ensured that only imaging data prior to diagnosis were used to predict dementia conversion. Continuous risk factors, such as blood pressure, were averaged over all time points. Smoking status was defined as smoking during the majority of follow-up, and diabetes as presence at any point during follow-up. A stepwise multivariate Cox regression model including all annualized MRI change rates and risk factors, plus baseline age, premorbid IQ, and sex, was applied to identify independent predictors of dementia.

To assess classification performance of the regression model, we performed discriminant function analyses. First we identified the discriminant value of a conventional risk factor model including age, sex, premorbid IQ, and cardiovascular risk factors. Subsequently we compared significance, sensitivity and specificity, area under the receiver operating characteristic curve (AUC; equivalent to a C statistic),^25^ and model stability (i.e., through leave-one-out cross-validation) of that model to that of a model that included the significant variables from the multivariate Cox regression and their baseline values (the relationship between baseline MRI measures and cognition is not investigated in this article, but has been previously published for the 121 patients recruited^10^).

## RESULTS

### Conversion to dementia.

A total of 18 (18.2%) of 99 patients converted to dementia during the 5-year follow-up. Dementia diagnosis was based on clinical diagnosis (n = 8), review of medical records (n = 3), and meeting dementia thresholds for MMSE and IADL scores (n = 7). Mean (SD) time to dementia conversion was 3.31 ± 1.40 years. Deaths and other endpoints during follow-up are shown in appendix e-4.

### Change in MRI and cognitive measures.

Over the 3-year imaging period, there was an increase in WMH lesion load, worsening of WM tissue microstructure (decreased MD-NPH), and decreased brain volume (table 1). Seventy-four new lacunes were observed in 27 patients. Nineteen developed 1–2 lacunes, and 8 ≥3 (maximum 9). A total of 173 new CMB occurred in 35 individuals; 10 developed a single CMB, 14 developed 2–5 CMB, and 11 developed ≥6 CMB.

**Figure 1:**
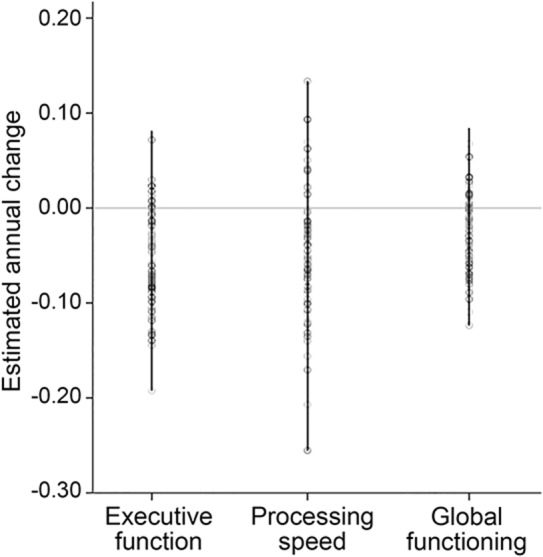
Estimated annual change rates for cognitive indices The dot lines show all estimated individual annual progression rates of executive function, processing speed, and global functioning, as modeled using linear mixed effect models over all available time points.

During 5-year cognitive follow-up, there was a statistically significant decline in EF, PS, and global functioning (table 1). No change in working and long-term memory was observed. We therefore limited further analyses to EF, PS, and global functioning. Cognitive change varied markedly between individual patients, with some showing marked decline and others no decline. Estimated annualized progression rates are shown in figure 1.

### Relationship between imaging change and cognition change.

Univariate models showed decline in EF was associated with lower baseline premorbid IQ (β = 0.219, *p* = 0.026), greater change of MD-NPH (β = 0.275, *p* = 0.007), and new lacunes (β = −0.269, *p* = 0.003) but not brain volume (β = −0.030, *p* = 0.773) or CMB (β = −0.190, *p* = 0.061) or WMH (β = 0.077, *p* = 0.449). In contrast, no neuroimaging marker, age, IQ, or sex correlated with decline in PS. Decline in global functioning was associated with lower premorbid IQ (β = 0.307, *p* = 0.002), greater change in MD-NPH (β = 0.262, *p* = 0.011), new lacunes (β = −0.265, *p* = 0.004), and new CMB (β = −0.218, *p* = 0.035) but not WMH (β = 0.677, *p* = 0.500) or brain volume (β = −0.113, *p* = 0.277).

Table 2 shows standardized regression coefficients from multivariate models investigating which MRI markers correlated with cognitive decline. New lacunes, decline in MD-NPH, and premorbid IQ were independently associated with decline in EF. The model explained 17.9% of variance in EF change (*F*_3,91_ = 6.63, *p* < 0.001). Age, premorbid IQ, change in MD-NPH, and new lacunes were all independent correlates of decline in global functioning, with the model explaining 26.6% of variance (*F*_4,88_ = 7.98, *p* < 0.001).

**Table 2:**
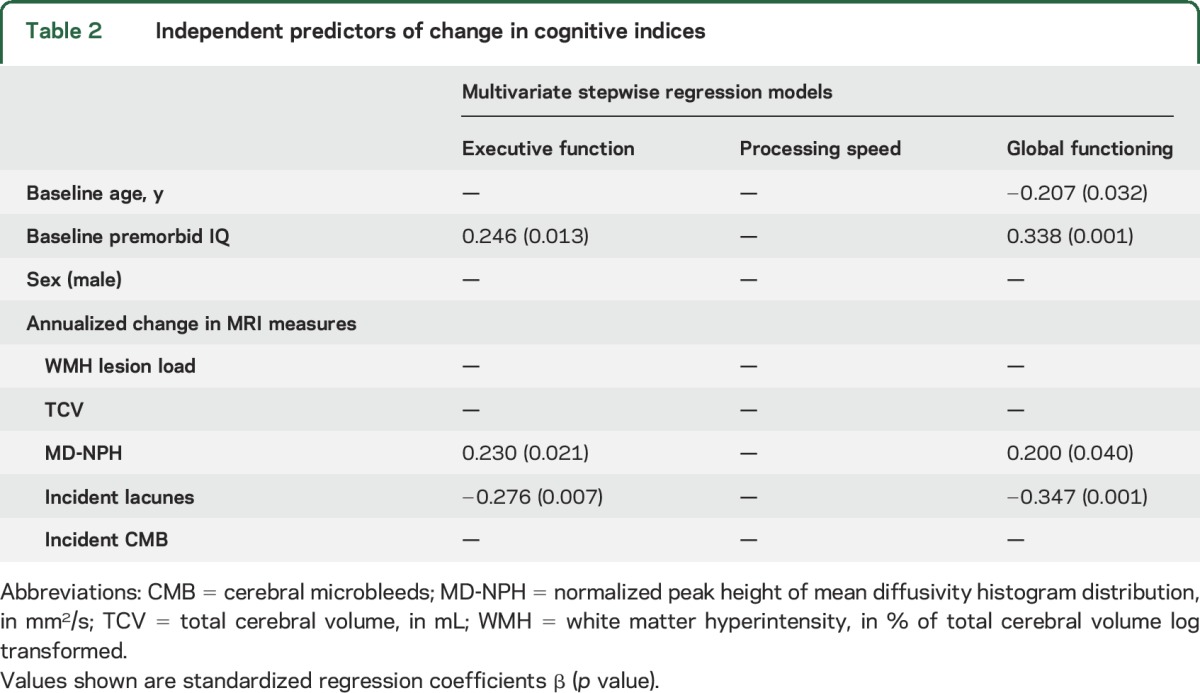
Independent predictors of change in cognitive indices

### Imaging predictors of conversion to dementia.

Univariate Cox regression analyses revealed only greater MD-NPH change (hazard ratio 0.004; *p* = 0.034) was indicative of conversion to dementia (table 3).

**Table 3:**
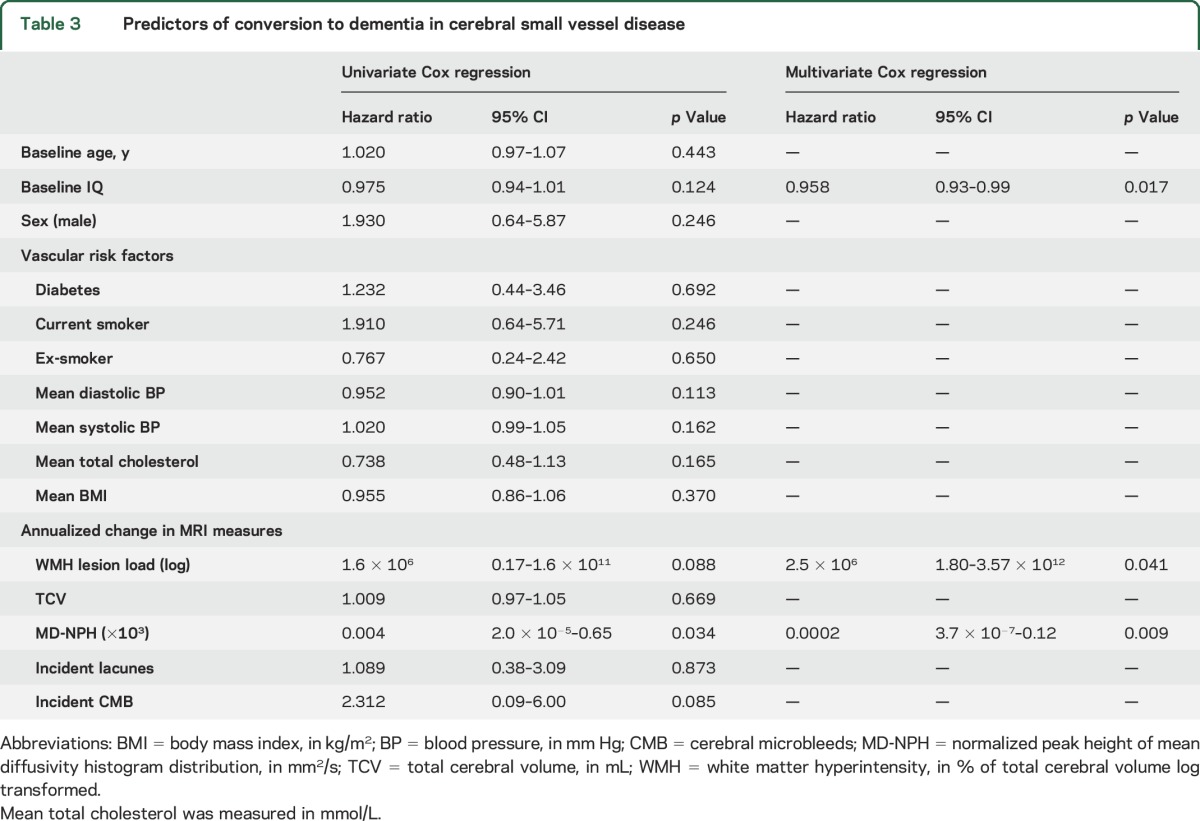
Predictors of conversion to dementia in cerebral small vessel disease

On multivariate Cox regression including all MRI measures, as well as age, sex, IQ, and vascular risk factors, only higher premorbid IQ, greater increase in WMH, and greater decrease in MD-NPH independently predicted conversion to dementia (table 3).

### Dementia prediction accuracy.

Discriminant analysis in which age, sex, and premorbid IQ and vascular risk factors were entered did not discriminate between dementia converters and nonconverters (Wilks lambda [*df* 9] = 0.865, χ^2^ = 13.43, *p* = 0.144). In contrast, discriminant analysis including the MRI variables found to be associated with progression to dementia in the previous multivariate Cox regression (i.e., change in MD-NPH, WMH, and premorbid IQ) and their MRI baseline values was significant (Wilks lambda [*df* 5] = 0.759, χ^2^ = 24.64; *p* < 0.001). It correctly classified 80.9% of patients with 80.0% sensitivity and 81.0% specificity and achieved an AUC of 0.849, corresponding to a C statistic of 0.849. After leave-one-out cross-validation, this model remained stable, with a sensitivity of 73.3% and specificity of 78.5%.

## DISCUSSION

In this longitudinal prospective study in symptomatic SVD, change in multimodal MRI measures over a 3-year period, during which little change in cognition was detectable, predicted long-term cognitive decline and dementia. In particular, diffuse WM damage on DTI predicted decline in EF and progression to dementia. It was striking that while some patients had marked cognitive decline, others did not decline. This emphasizes the need for predictive tools to identify those who are likely to develop dementia both for individual risk prediction and to identify who may benefit from specific treatments. Our results suggest that MRI may be useful in risk prediction.

Previous cross-sectional studies using DTI have demonstrated abnormalities not only within WMH,^26^ but also in NAWM,^8^ and have shown these DTI measures correlate with cognition more strongly than WMH volume.^27,28^ This finding, indicating that diffuse WM damage is associated with cognitive decline, has led to the hypothesis that disruption of WM tracks and secondary disconnection of complex cortical–subcortical networks causes cognitive impairment. However, cross-sectional studies demonstrate association, but cannot prove causality. Longitudinal studies provide stronger evidence that associations are causal. This study provides some of the first evidence that change in DTI measures correlates with subsequent cognitive decline in SVD, and therefore provides support for the hypothesis that diffuse WM damage on DTI causes cognitive impairment.

Using discriminant analysis, we showed that WM microstructure in addition to WMH lesion load and premorbid IQ had significant power to discriminate dementia converters from nonconverters with a C statistic of 0.849. This is a level of prediction that would be clinically useful and is, for example, higher than the 0.81 previously reported for an all-dementia model, which included MRI variables in addition to demographic, vascular risk factor, and cognitive variables.^29^ Replication in further independent SVD test populations is needed.

Our results provide further support for the validity of MRI measures as surrogate markers in clinical trials evaluating new treatments for SVD. Currently there are few effective treatments for patients with SVD-related cognitive decline. A major obstacle to assessing new treatments is the lack of detectable longitudinal change in cognition over short time periods.^3,7^ This has led to the suggestion that MRI measures may be useful to assess new therapies.^7,30^ Using MRI markers could markedly reduce sample sizes required to detect treatment effects.^7^ However, before such a surrogate marker is adopted clinically, it is essential to show that change in the marker correlates with change in clinical endpoints. Our study provides some of the first data that this is indeed the case with change in MD-NPH, the DTI measure most sensitive to change,^20^ correlating with eventual cognitive decline and progression to dementia.

Previous cross-sectional studies in SVD have shown that, in addition to WM damage imaged on DTI, lacunar infarcts^31,32^ and brain volume^9,33^ are also associated with cognitive impairment, while inconsistent associations have been shown for CMB.^31,34,35^ In this study, new lacunar infarcts were associated with decline in EF. Although 74 new lacunar infarcts were detected over 3 years of imaging follow-up, only 3 patients had symptomatic lacunar strokes in this period. Therefore, although the vast majority of new lacunar infarcts are apparently asymptomatic, they are associated with cognitive decline. It has been hypothesized that lacunar infarcts do so by causing previously mentioned disconnection.^32^ Support for this has been provided by cross-sectional data showing that network efficiency mediates the association of lacunar infarcts on cognitive impairment.^36^ In contrast to previous cross-sectional data, we did not find any association between brain volume and cognitive decline or dementia despite a detectable decline in volume over time.

Previous studies have shown little or no cognitive change over shorter time periods in this patient group.^4^ An analysis of cognition at 3 years in the same SCANS study showed minimal detectable change,^4^ and in the large SPS3 trial in MRI-confirmed lacunar stroke no change was detected over a 2-year period either.^3^ The current study shows that with longer follow-up, change is detectable. The lack of cognitive change over shorter periods of time is likely to be a reflection of testing methodology (i.e., variability in test results and learning effects), as well as the slow rate of cognitive decline in some individuals.

In contrast to associations we found between DTI measures and EF, we found no association with decline in PS. This is perhaps surprising in light of the association between PS and MRI measures in previous cross-sectional studies.^10,37^ This may reflect the relatively large motor performance component in our PS measure leading to reduced specificity. Alternatively, the mechanisms underlying PS impairment in SVD might differ from those causing EF impairment. PS impairment has been shown to associate with global efficiency derived from DTI tractography networks,^14^ and may be a consequence of more diffuse network disruption. Decline in PS may thus be better described by alternative analysis methods such as network analysis.^36^

The present study had a number of limitations. First, it suffered from moderate data loss during follow-up, although the dropout rate is comparable to longitudinal aging studies.^38^ As reported previously, patients without complete follow-up tended to be older and more disabled,^4,7^ which may have led to an underestimation of MRI and cognitive progression rates. Second, all MRI data were acquired on a 1.5T scanner. Although the same scanner was used, which was not upgraded during the duration of the study, image data quality could be improved by higher field strengths and spatial resolution with isotropic voxel dimensions.^39^ SVD represents a spectrum of disease from asymptomatic WMH in community populations through to patients with multiple lacunes and extensive WMH who present with vascular dementia.^1^ This study investigated a population with moderate to severe symptomatic SVD who have a higher risk of progressing to dementia. In this specific patient group, we showed that a significant proportion progresses to dementia over 5 years. Our results now require replication in patients with less severe SVD, such as in community studies, in which WM DTI measures have been shown to correlate with cognition cross-sectionally.^27,40^

## Supplementary Material

Data Supplement

## GLOSSARY

AUC: area under the receiver operating characteristic curve
CMB: cerebral microbleeds
DSM-5: *Diagnostic and Statistical Manual of Mental Disorders, 5th edition*
DTI: diffusion tensor imaging
EF: executive function
IADL: Instrumental Activities of Daily Living
LME: linear mixed effect
MD: mean diffusivity
MMSE: Mini-Mental State Examination
NAWM: normal-appearing white matter
NPH: normalized peak height
PS: processing speed
SCANS: St George's Cognition and Neuroimaging in Stroke
SVD: small vessel disease
WM: white matter
WMH: white matter hyperintensities

## References

[1] Pantoni L. Cerebral small vessel disease: from pathogenesis and clinical characteristics to therapeutic challenges. Lancet Neurol 2010;9:689–701. 20610345 10.1016/S1474-4422(10)70104-6

[2] Debette S, Markus HS. The clinical importance of white matter hyperintensities on brain magnetic resonance imaging: systematic review and meta-analysis. BMJ 2010;341:c3666. 20660506 10.1136/bmj.c3666 PMC2910261

[3] Pearce LA, McClure LA, Anderson DC, et al. Effects of long-term blood pressure lowering and dual antiplatelet treatment on cognitive function in patients with recent lacunar stroke: a secondary analysis from the SPS3 randomised trial. Lancet Neurol 2014;13:1177–1185. 25453457 10.1016/S1474-4422(14)70224-8 PMC4284947

[4] Lawrence AJ, Brookes RL, Zeestraten EA, Barrick TR, Morris RG, Markus HS. Pattern and rate of cognitive decline in cerebral small vessel disease: a prospective study. PLoS One 2015;10:e0135523. 26273828 10.1371/journal.pone.0135523 PMC4537104

[5] Patel B, Markus HS. Magnetic resonance imaging in cerebral small vessel disease and its use as a surrogate disease marker. Int J Stroke 2011;6:47–59. 21205241 10.1111/j.1747-4949.2010.00552.x

[6] Wardlaw JM, Smith EE, Biessels GJ, et al. Neuroimaging standards for research into small vessel disease and its contribution to ageing and neurodegeneration. Lancet Neurol 2013;12:822–838. 23867200 10.1016/S1474-4422(13)70124-8 PMC3714437

[7] Benjamin P, Zeestraten EA, Lambert C, et al. Progression of MRI markers in cerebral small vessel disease: sample size considerations for clinical trials. J Cereb Blood Flow Metab 2016;36:228–240. 26036939 10.1038/jcbfm.2015.113 PMC4758545

[8] O'Sullivan M, Summers PE, Jones DK, Jarosz JM, Williams SCR, Markus HS. Normal-appearing white matter in ischemic leukoaraiosis: a diffusion tensor MRI study. Neurology 2001;57:2301–2310. 11756617 10.1212/wnl.57.12.2307

[9] Nitkunan A, Lanfranconi S, Charlton RA, Barrick TR, Markus HS. Brain atrophy and cerebral small vessel disease: a prospective follow-up study. Stroke 2011;42:133–138. 21148440 10.1161/STROKEAHA.110.594267

[10] Lawrence AJ, Patel B, Morris RG, et al. Mechanisms of cognitive impairment in cerebral small vessel disease: multimodal MRI results from the St George's Cognition and Neuroimaging in Stroke (SCANS) study. PLoS One 2013;8:e61014. 23613774 10.1371/journal.pone.0061014 PMC3632543

[11] Carey CL, Kramer JH, Josephson SA, et al. Subcortical lacunes are associated with executive dysfunction in cognitively normal elderly. Stroke 2008;39:397–402. 18096844 10.1161/STROKEAHA.107.491795 PMC2443738

[12] Jokinen H, Lipsanen J, Schmidt R, et al. Brain atrophy accelerates cognitive decline in cerebral small vessel disease: the LADIS study. Neurology 2012;78:1785–1792. 22592361 10.1212/WNL.0b013e3182583070

[13] O'Sullivan M, Jones DK, Summers PE, Morris RG, Williams SCR, Markus HS. Evidence for cortical “disconnection” as a mechanism of age-related cognitive decline. Neurology 2001;57:632–638. 11524471 10.1212/wnl.57.4.632

[14] Lawrence AJ, Chung A, Morris RG, Markus HS, Barrick TR. Structural network efficiency is associated with cognitive impairment in small vessel disease. Neurology 2014;44:1–24. 10.1212/WNL.0000000000000612 PMC4115608 24951477

[15] Bamford J, Sandercock P, Jones L, Warlow C. The natural history of lacunar infarction: the Oxfordshire Community Stroke Project. Stroke 1987;18:545–551. 3590244 10.1161/01.str.18.3.545

[16] Fazekas F, Chawluk JB, Alavi A, Hurtig HI, Zimmerman RA. MR signal abnormalities at 1.5 T in Alzheimer's dementia and normal aging. Am J Roentgenol 1987;149:351–356. 3496763 10.2214/ajr.149.2.351

[17] Linn J, Halpin A, Demaerel P, et al. Prevalence of superficial siderosis in patients with cerebral amyloid angiopathy. Neurology 2010;74:1346–1350. 20421578 10.1212/WNL.0b013e3181dad605 PMC2875936

[18] Lambert C, Benjamin P, Zeestraten EA, Lawrence AJ, Barrick TR, Markus HS. Longitudinal patterns of leukoaraiosis and brain atrophy in symptomatic small vessel disease. Brain 2016;139:1136–1151. 26936939 10.1093/brain/aww009 PMC4806220

[19] Cordonnier C, Potter GM, Jackson CA, et al. Improving interrater agreement about brain microbleeds: development of the brain observer microbleed scale (BOMBS). Stroke 2009;40:94–99. 19008468 10.1161/STROKEAHA.108.526996

[20] Zeestraten EA, Benjamin P, Lambert C, et al. Application of diffusion tensor imaging parameters to detect change in longitudinal studies in cerebral small vessel disease. PLoS One 2016;11:e0147836. 26808982 10.1371/journal.pone.0147836 PMC4726604

[21] American Psychiatric Association. Diagnostic and Statistical Manual of Mental Disorders, 5th ed (DSM-5). Washington, DC: American Psychiatric Association; 2013.

[22] Tombaugh TN, McIntyre NJ. The Mini-Mental State Examination: a comprehensive review. J Am Geriatr Soc 1992;40:922–935. 1512391 10.1111/j.1532-5415.1992.tb01992.x

[23] Barberger-Gateau P, Commenger D, Gagnon M, Letenneur L, Sauvel C, Dartigues J-F. Instrumental Activities of Daily Living as a screening tool for cognitive impairment and dementia in elderly community dwellers. J Am Geriatr Soc 1992;40:1129–1134. 1401698 10.1111/j.1532-5415.1992.tb01802.x

[24] Rasbash J, Charlton C, Browne WJ, Healy M, Cameron B. MLwiN Version 2.1. Centre for Multilevel Modelling. Bristol: University of Bristol; 2009.

[25] Hanley JA, McNeil BJ. The meaning and use of the area under a receiver operating characteristic (ROC) curve. Radiology 1982;143:29–36. 7063747 10.1148/radiology.143.1.7063747

[26] Jones DK, Lythgoe D, Horsfield MA, Simmons A, Williams SCR, Markus HS. Characterization of white matter damage in ischemic leukoaraiosis with diffusion tensor MRI. Stroke 1999;30:393–397. 9933277 10.1161/01.str.30.2.393

[27] della Nave R, Foresti S, Pratesi A, et al. Whole-brain histogram and voxel-based analyses of diffusion tensor imaging in patients with leukoaraiosis: correlation with motor and cognitive impairment. AJNR Am J Neuroradiol 2007;28:1313–1319. 17698534 10.3174/ajnr.A0555 PMC7977673

[28] Nitkunan A, Barrick TR, Charlton RA, Clark CA, Markus HS. Multimodal MRI in cerebral small vessel disease: its relationship with cognition and sensitivity to change over time. Stroke 2008;39:1999–2005. 18436880 10.1161/STROKEAHA.107.507475

[29] Barnes DE, Covinsky KE, Whitmer RA, Kuller LH, Lopez OL, Yaffe K. Predicting risk of dementia in older adults: the late-life dementia risk index. Neurology 2009;73:173–179. 19439724 10.1212/WNL.0b013e3181a81636 PMC2715571

[30] Schmidt R, Seiler S, Loitfelder M. Longitudinal change of small-vessel disease-related brain abnormalities. J Cereb Blood Flow Metab 2016;36:1–8. 10.1038/jcbfm.2015.72 PMC4758559 25899293

[31] Viswanathan A, Gschwendtner A, Guichard J-P, et al. Lacunar lesions are independently associated with disability and cognitive impairment in CADASIL. Neurology 2007;69:172–179. 17620550 10.1212/01.wnl.0000265221.05610.70

[32] Benjamin P, Lawrence AJ, Lambert C, et al. Strategic lacunes and their relationship to cognitive impairment in cerebral small vessel disease. Neuroimage Clin 2014;4:828–837. 24936433 10.1016/j.nicl.2014.05.009 PMC4055894

[33] Viswanathan A, Godin O, Jouvent E, et al. Impact of MRI markers in subcortical vascular dementia: a multi-modal analysis in CADASIL. Neurobiol Aging 2010;31:1629–1636. 18926602 10.1016/j.neurobiolaging.2008.09.001

[34] Patel B, Lawrence AJ, Chung AW, et al. Cerebral microbleeds and cognition in patients with symptomatic small vessel disease. Stroke 2013;44:356–361. 23321452 10.1161/STROKEAHA.112.670216

[35] Werring DJ, Frazer DW, Coward LJ, et al. Cognitive dysfunction in patients with cerebral microbleeds on T2*-weighted gradient-echo MRI. Brain 2004;127:2265–2275. 15282216 10.1093/brain/awh253

[36] Tuladhar AM, van Dijk E, Zwiers MP, et al. Structural network connectivity and cognition in cerebral small vessel disease. Hum Brain Mapp 2016;37:300–310. 26466741 10.1002/hbm.23032 PMC6867512

[37] Schmidt R, Ropele S, Ferro J, et al. Diffusion-weighted imaging and cognition in the leukoariosis and disability in the elderly study. Stroke 2010;41:402–408. 20203319 10.1161/STROKEAHA.109.576629

[38] Glymour MM, Chěne G, Tzourio C, Dufouil C. Brain MRI markers and dropout in a longitudinal study of cognitive aging: the Three-City Dijon Study. Neurology 2012;79:1340–1348. 22972647 10.1212/WNL.0b013e31826cd62a PMC3448743

[39] Vrenken H, Jenkinson M, Horsfield MA, et al. Recommendations to improve imaging and analysis of brain lesion load and atrophy in longitudinal studies of multiple sclerosis. J Neurol 2013;260:2458–2471. 23263472 10.1007/s00415-012-6762-5 PMC3824277

[40] Hannesdottir K, Nitkunan A, Charlton RA, Barrick TR, MacGregor GA, Markus HS. Cognitive impairment and white matter damage in hypertension: a pilot study. Acta Neurol Scand 2009;119:261–268. 18798828 10.1111/j.1600-0404.2008.01098.x

