# Supplementary material for: Change in multimodal MRI markers predicts dementia risk in cerebral small vessel disease
Source: Neurology. 2017 Oct 31;89(18):1869–76. doi: 10.1212/WNL.0000000000004594 (PMC5664300; doi:10.1212/WNL.0000000000004594)
Supplement: Data Supplement [file supp_89_18_1869__index.html]

Change in multimodal MRI markers predicts dementia risk in cerebral small vessel disease — Data Supplement 

# Change in multimodal MRI markers predicts dementia risk in cerebral small vessel disease

## Data Supplement

**Neurology® data supplements are not copyedited before publication. Published editorials and translations have been copyedited.  
 © 2017 American Academy of Neurology.  
  
 Files in this Data Supplement:**

- Figure e-1 - PDF
- Appendix e-1 - Microsoft Word file
- Appendix e-2 - Microsoft Word file
- Appendix e-3 - Microsoft Word file
- Appendix e-4 - Microsoft Word file
- e-References - Microsoft Word file
- Table e-1 - Microsoft Word file
- Table e-2 - Microsoft Word file
