# Supplementary material for: Change in multimodal MRI markers predicts dementia risk in cerebral small vessel disease
Source: Neurology. 2017 Oct 31;89(18):1869–76. doi: 10.1212/WNL.0000000000004594 (PMC5664300; doi:10.1212/WNL.0000000000004594)
Supplement: Data Supplement [file supp_WNL.0000000000004594_Figure_e-1.pdf]

Figure e-1. Mean diffusivity histogram distribution in all white matter tissue.

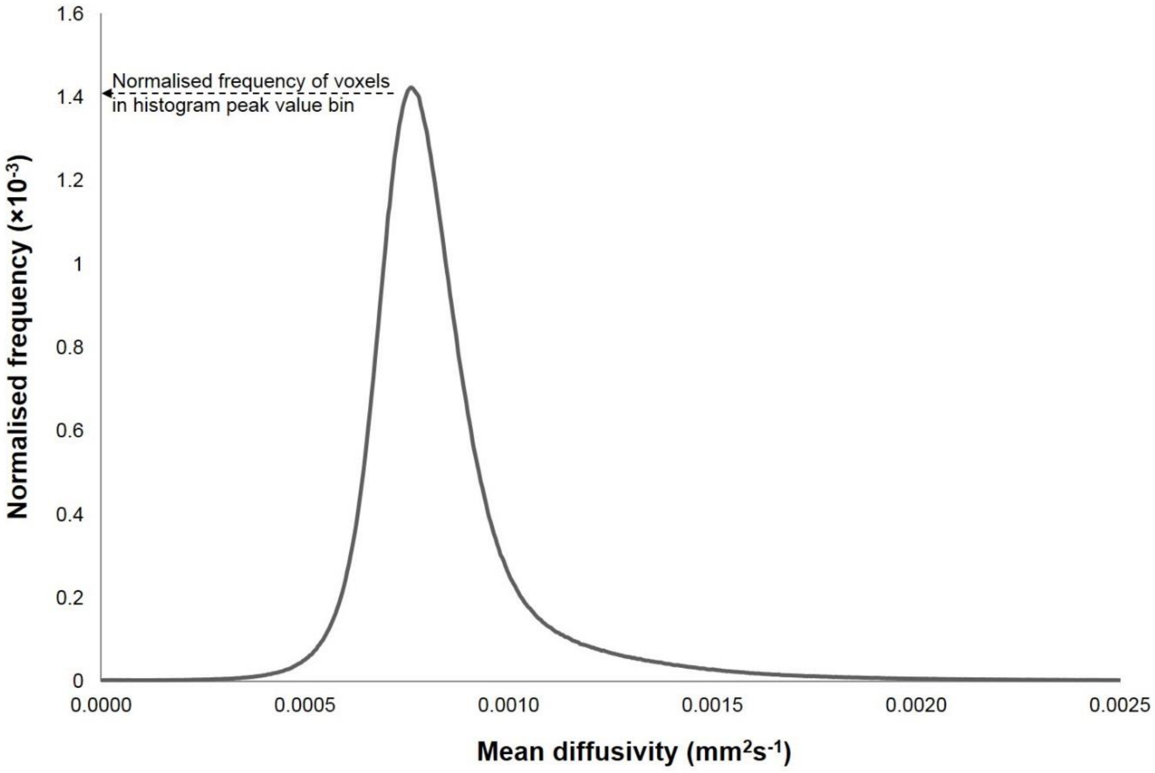

Figure e-1. Depiction of normalised peak height of mean diffusivity histogram distribution in all white matter tissue. The normalised peak height is the frequency of voxels with the histogram peak value. Over time with an increase in microstructural tissue damage, mean diffusivity increases. This results in a wider spread of high diffusivity values which are observed with greater frequency, increasing the peak value and lowering the frequency with which that peak value is observed, thus lowering the normalised peak height.
