# Supplementary material for: Change in multimodal MRI markers predicts dementia risk in cerebral small vessel disease
Source: Neurology. 2017 Oct 31;89(18):1869–76. doi: 10.1212/WNL.0000000000004594 (PMC5664300; doi:10.1212/WNL.0000000000004594)
Supplement: Data Supplement [file supp_WNL.0000000000004594_Appendix_e-1.docx]

**Appendix e-1**

# Methods

***MRI acquisition***

MR images were acquired using a 1.5-Tesla GE Signa HDxt system (General Electric, Milwaukee, WI, USA) with maximum gradient amplitude of 33 mT/m and a proprietary head coil. To standardise head position, patients were placed in a neutral position in the head coil with an alignment marker at the nasal bridge. To minimise head movement foam pads and a Velcro strap across the forehead were used. Total imaging time was approximately 45 minutes during which the following scan sequences, all providing whole head coverage, were obtained: 3D T1-weighted spoiled gradient recalled echo, FLAIR, T2*-weighted gradient recalled echo and single shot spin echo planar diffusion-weighted imaging. All acquired sequences provided whole head coverage using a field of view of 240x240 mm^2^.

1. 3D T1-weighted spoiled gradient recalled echo: TR=11.5ms, TE=5ms, 176 slices without slice-gap, matrix = 256×192, flip angle=18°, providing 1.1mm^3^ isotropic voxels;
2. Fluid attenuated inversion recovery (FLAIR): TR=9000ms, TE=130ms, TI=2200ms, 28 5mm slices without slice-gap, matrix = 256×192;
3. T2*-weighted gradient recalled echo: TR=300ms, TE=30ms, 28 5mm slices without slice-gap, acquisition matrix=256×192 mm^2^, flip angle=15°
4. Single shot spin echo planar diffusion-weighted imaging: TR=15600ms, TE=93.4ms, 55 slices without slice-gap with 2.5mm^3^ isotropic voxels, matrix=96×96, 8 non-diffusion-weighted images (b=0smm^-2^) followed by diffusion-weighted volumes with diffusion gradients applied (b=1000smm^-2^) in 25 non-collinear directions and the negative of these.
