# Supplementary material for: Change in multimodal MRI markers predicts dementia risk in cerebral small vessel disease
Source: Neurology. 2017 Oct 31;89(18):1869–76. doi: 10.1212/WNL.0000000000004594 (PMC5664300; doi:10.1212/WNL.0000000000004594)
Supplement: Data Supplement [file supp_WNL.0000000000004594_Appendix_e-2.docx]

**Appendix e-2**

# Methods

***Conventional MRI markers of small vessel disease***

*Total cerebral volume:* Total cerebral volume (TCV) at each time point was defined as the sum of grey matter, normal appearing WM and WMH volumes as calculated in subject space from the binarised segmentation maps. Changes in TCV were quantified in millilitres (ml).

*WMH lesion load:* The volume of WMH is expressed as a percentage ratio between TCV and WMH volume (WMH lesion load^e1^). Weighting according to brain-size is done as normalising according to total intracranial volume^e2^ poses a problem in longitudinal studies where there may be no WMH growth but ongoing all WM atrophy. In that case using total WMH volume or normalising to intracranial volume would incorrectly show that the lesions are less severe over time. This problem is negated by normalising according to brain volume, which will also factor in brain atrophy thereby providing a more accurate reflection of disease severity.

*Lacunes:* A consultant neuroradiologist (ADM) evaluated T1-weighted, T2-weighted and FLAIR scans for lacunes. To ensure an underlying SVD aetiology of lacunes and exclude perivascular spaces, lacunes were defined as a CSF filled cavities of 3-15 mm in diameter with a surrounding rim of FLAIR hyperintensity^e3^. Follow-up scans for each patient were registered to a group average template, as described in Lambert et al., (2016) ^e4^, and assessed in succession of each other to enable accurate identification of incident lacunes. The presence and number of incident lacunes were noted for each patient. Lacune number reliability metrics were checked using all available scans of a subset of 10 randomly selected patients assessed by the consultant neuroradiologist and an additional rater (EAZ). The intra-class correlation coefficient was 0.99.

*Cerebral microbleeds:* CMB were defined as homogeneous round focal areas < 10 mm in diameter of low signal intensity on T2*-weighted GRE images. Only CMB meeting the Brain Observer Microbleed Rating Scale (BOMBS) ^e5^ criteria for “certain” CMB were analysed. Presence and number of new CMB were noted for all patients. All baseline CMB were identified by a single consultant neuroradiologist. CMB on follow-up were identified by a single rater (EAZ). CMB number reliability metrics were checked using all available scans of a subset of 10 randomly selected patients assessed by both raters. The intra-class correlation coefficient was 0.99.
