# Supplementary material for: Change in multimodal MRI markers predicts dementia risk in cerebral small vessel disease
Source: Neurology. 2017 Oct 31;89(18):1869–76. doi: 10.1212/WNL.0000000000004594 (PMC5664300; doi:10.1212/WNL.0000000000004594)
Supplement: Data Supplement [file supp_WNL.0000000000004594_Appendix_e-3.docx]

**Appendix e-3**

# Methods

***Diffusion Pre-processing***

Following realignment of diffusion-weighted images to remove eddy current distortions using the FSL Linear Image Registration Tool (FLIRT, FMRIB Software Library, [www.fmrib.ox.ac.uk/fsl](http://www.fmrib.ox.ac.uk/fsl)) ^e6^, the acquired positive and negative diffusion gradient direction images (b = 1000 smm^-2^) were geometrically averaged to eliminate gradient cross-terms^e7^. The eight images without diffusion weighting *b* = 0 smm^-2^) were co-registered and averaged to give a T2-weighted echo planar image, henceforth referred to the *b*0 image. Diffusion tensor maps were computed using FSL DTIfit and MD maps were calculated.

To obtain tissue class mask images in diffusion space, an affine transformation, followed by non-linear transformation, was computed for each T1-weighted image to the *b*0 image using FMRIB Non-linear Image Registration Tool (FNIRT) ^e8^. These transformations were subsequently applied to all tissue segmentations to map them to the diffusion data. Tissue class masks included voxels with probabilities of GM, NAWM or WMH > 0.5. Based on a diffusivity threshold, spurious CSF voxels were removed from the tissue masks (i.e. voxels with MD > 0.0026 mm^2^s^-1^ were considered to contain CSF and excluded). For this study, the histogram distribution of mean diffusivity in normal appearing WM plus WMH, termed all WM, was evaluated (range 0-0.004 mm^2^s^-1^, bin width 0.000004 mm^2^s^-1^). To account for differences in brain volume, histograms were normalised by the number of voxels present in the all WM mask. The normalised frequency of voxels with the histogram peak value in all WM tissue, the normalised peak height, was used as a measure of tissue microstructure over time as we have shown it is the most stable and sensitive DTI measure of change^e9^.
