# Supplementary material for: Change in multimodal MRI markers predicts dementia risk in cerebral small vessel disease
Source: Neurology. 2017 Oct 31;89(18):1869–76. doi: 10.1212/WNL.0000000000004594 (PMC5664300; doi:10.1212/WNL.0000000000004594)
Supplement: Data Supplement [file supp_WNL.0000000000004594_Appendix_e-4.docx]

**Appendix e-4**

# Results

## ***Clinical endpoints during follow-up***

Over the 5-year course of the study, eight of 99 patients died. Causes of death were: cancer (n=1), respiratory (n=1), intracerebral haemorrhages (n=2), other health related (n=2), interpersonal violence (n=1) and unknown (n=1).

Seven patients exited the study after reaching clinical endpoints and were from there onwards excluded from follow-up over time: clinical dementia diagnosis in 4, intracerebral haemorrhage in 2, and major cognitive impairment related to hypoxia following a cardiac arrest in 1. Four patients suffered a new symptomatic lacunar stroke during follow-up (3 of which were during the three years of imaging follow-up) and according to the study protocol were eligible to remain in the study; three of these, however, subsequently dropped out due to disability.
