# Supplementary material for: Change in multimodal MRI markers predicts dementia risk in cerebral small vessel disease
Source: Neurology. 2017 Oct 31;89(18):1869–76. doi: 10.1212/WNL.0000000000004594 (PMC5664300; doi:10.1212/WNL.0000000000004594)
Supplement: Data Supplement [file supp_WNL.0000000000004594_e-References.docx]

# e-References

e1. Benjamin P, Lawrence AJ, Lambert C, et al. Strategic lacunes and their relationship to cognitive impairment in cerebral small vessel disease. NeuroImage Clin. 2014 Jan;4:828–837.

e2. Viswanathan A, Godin O, Jouvent E, et al. Impact of MRI markers in subcortical vascular dementia: a multi-modal analysis in CADASIL. Neurobiol Aging. 2010 Sep;31(9):1629–1636.

e3. Wardlaw JM, Smith EE, Biessels GJ, et al. Neuroimaging standards for research into small vessel disease and its contribution to ageing and neurodegeneration. Lancet Neurol. Elsevier Ltd; 2013 Aug;12(8):822–838.

e4. Lambert C, Benjamin P, Zeestraten EA, Lawrence AJ, Barrick TR, Markus HS. Longitudinal patterns of leukoaraiosis and brain atrophy in symptomatic small vessel disease. Brain. 2016;139:1136–1151.

e5. Cordonnier C, Potter GM, Jackson CA, et al. Improving interrater agreement about brain microbleeds: development of the Brain Observer MicroBleed Scale (BOMBS). Stroke. 2009 Jan;40(1):94–99.

e6. Jenkinson M, Smith S. A global optimisation method for robust affine registration of brain images. Med Image Anal. 2001 Jun;5(2):143–156.

e7. Neeman M, Freyer JP, Sillerud LO. A simple method for obtaining cross-term-free images for diffusion anisotropy studies in NMR microimaging. Magn Reson Med. 1991 Sep;21(1):138–143.

e8. Andersson JLR, Jenkinson M, Smith S. Non-linear registration aka Spatial normalisation. FMRIB Technical Report TR07JA2. FMRIB Tech Rep TR07JA2. 2007.

e9. Zeestraten EA, Benjamin P, Lambert C, et al. Application of diffusion tensor imaging parameters to detect change in longitudinal studies in cerebral small vessel disease. PLoS One. 2016;11(1):e0147836.

e10. Wechsler D. Wechsler Memory Scale. Third edition manual. San Antonio, TX: The Psychological Corporation; 1997.

e11. Klove H. Clinical neuropsychology. In: Forster FM, editor. Med Clin North Am. New York: Saunders; 1963.

e12. Mitrushina M, Boone KB, Razani J, D’Elia LF. Handbook of Normative Data for Neuropsychological Assessment. 2nd ed. New York: Oxford University Press; 2005.

e13. Coughlan AK, Oddy M, Crawford JR. The BIRT Memory and Information Processing Battery (B-MIPB). Wakefield, UK: The Brain Injury Rehabiliation Trust (BIRT); 2007.

e14. Wechsler D. Wechsler Adult Intelligence Scale-III. San Antonio, TX: The Psychological Corporation; 1997.

e15. Delis DC, Kaplan E, Kramer JH. Delis-Kaplan Executive Function Scale. San Antonio, TX: The Psychological Corporation; 2001.

e16. Nelson HE. A modified card sorting test sensitive to frontal lobe defects. Cortex. 1976;12:313–324.

e17. Nagahama Y, Okina T, Suzuki N, et al. Factor structure of a modified version of the wisconsin card sorting test: an analysis of executive deficit in Alzheimer’s disease and mild cognitive impairment. Dement Geriatr Cogn Disord. 2003;16(2):103–112.

e18. Reitan RM. The validity of the Trail Making Test as an indicator of organic brain damage. Percept Mot Skills. 1958;8:271–276.

e19. Nelson HE, Willison JR. National Adult Reading Test (NART): Test Manual. Second edi. NFER-Nelson; 1991.

e20. Folstein MF, Folstein SE, McHugh PR. Mini-Mental State: a practical method for grading the cognitive state of patients for the clinician. J Psychiatr Res. 1975;12(3):189–198.
