# Supplementary material for: Change in multimodal MRI markers predicts dementia risk in cerebral small vessel disease
Source: Neurology. 2017 Oct 31;89(18):1869–76. doi: 10.1212/WNL.0000000000004594 (PMC5664300; doi:10.1212/WNL.0000000000004594)
Supplement: Data Supplement [file supp_WNL.0000000000004594_Table_e-1.docx]

Table e-1.

Overview of the available neuroimaging and cognition data for each year of follow-up

|  | Complete neuroimaging available | Valid cognition available |
| --- | --- | --- |
| Baseline | 115 | 121 |
| Year 1 follow-up | 94^a^ | 96^b^ |
| Year 2 follow-up | 74 | 77 |
| Year 3 follow-up | 68 | 72 |
| Year 4 follow-up | n/a | 37 |
| Year 5 follow-up | n/a | 59 |

Table e-1. Complete neuroimaging data consists of conventional MRI (i.e. T1-weighted, T2*-weighted GRE and FLAIR) and DTI. Valid cognition numbers include sporadic missing cognitive data (3.86% across 9 tasks) where parts of the test battery could not be completed on a given occasion due to for example time constraints, patient motivation, brief assessment performed or experimenter error.

^a^Five subjects did not complete full neuroimaging at year 1 follow-up but returned for year 2 and/or 3 of follow-up, giving a total of 99 subjects who completed multiple neuroimaging sessions.

^b^Seven subjects did not complete neuropsychological testing at year 1 follow-up, but returned for (at least) one other testing session at a later time-point, giving a total of 103 subjects with multiple available cognitive assessments.
