# Supplementary material for: Change in multimodal MRI markers predicts dementia risk in cerebral small vessel disease
Source: Neurology. 2017 Oct 31;89(18):1869–76. doi: 10.1212/WNL.0000000000004594 (PMC5664300; doi:10.1212/WNL.0000000000004594)
Supplement: Data Supplement [file supp_WNL.0000000000004594_Table_e-2.docx]

Table e-2. Neuropsychological test battery.

| *Cognitive index*  Task name | Task description  *Used task measure* | References and/or normative data | | | |
| --- | --- | --- | --- | --- | --- |
| Long term memory |  | | |  | |
| WMS-III Logical Memory | Immediate and delayed recall of short stories  *Average* of immediate and delayed story detail recall scores* | | | ^e10^ | |
| WMS-III Visual  Reproduction | Immediate and delayed reproduction of line drawings  *Average* of immediate and delayed reproduction scores* | | | ^e10^ | |
| Working memory |  | | |  | |
| WMS-III Digit Span:  forwards & backwards | Immediate recall of digit strings  *Sum of total forwards and backwards scores* | | | ^e10^ | |
| Processing speed |  | | |  | |
| Grooved Pegboard | Pick-up and targeted placement of shaped small pegs  *Completion time of best hand trial* | | ^e11, e12^ | | |
| BMIPB Speed of  Information Processing | Speeded cancellation of second highest number in line of five two-digit numbers  *Total correct item score adjusted for motor speed as measured in control task* | | | ^e13^ | |
| WAIS-III Digit Symbol-  Coding | Speeded transcoding of numbers to symbols  *Total correct item score* | | | ^e14^ | |
| Executive function |  | | |  | |
| Verbal Fluency: FAS task | Timed generation of words beginning with the letters FAS or BHR  *Total correct words* | | | ^e15^ | |
| Modified Wisconsin Card  Sorting Test | Card sorting test invoking mental flexibility and strategy shifting  *Average* of number of completed categories score and perseverative errors* | | | | ^e16, e17†^ |
| Trail Making Test: Part B | Speeded pen and paper sequencing task, alternating between numbers and letters  *Completion time* | | | ^e18^ | |
| Global functioning | Average of all task measures listed above | | |  | |
| Premorbid intelligence | National Adult Reading Test-restandardised (NART) | | | ^e19^ | |
| Dementia screening | Mini Mental State Examination | | | ^e20^ | |

Table S2. Abbreviations: WMS – Wechsler Memory Scale; BMIPB – Birt Memory and Information Processing Battery; *Average task measures are calculated as the mean average of the z-score transformed task component scores. †A lack of published age adjusted normative data available for the modified Wisconsin Card Sorting Test led us to scale this data relative to age and gender-matched published sample data.
